# Amelioration of Sarcoptic Mange-Induced Oxidative Stress and Growth Performance in Ivermectin-Treated Growing Rabbits Using Turmeric Extract Supplementation

**DOI:** 10.3390/ani11102984

**Published:** 2021-10-16

**Authors:** Salma H. Abu Hafsa, Haytham Senbill, Mohamed M. Basyony, Ayman A. Hassan

**Affiliations:** 1 Livestock Research Department, Arid Lands Cultivation Research Institute, City of Scientific Research and Technological Applications, New Borg El-Arab, Alexandria 21934, Egypt; 2 Department of Applied Entomology and Zoology, Faculty of Agriculture, Alexandria University, Alexandria 21545, Egypt; haytham.senbill@alexu.edu.eg; 3 Agricultural Research Center, Animal Production Research Institute, Giza 12619, Egypt; mohamed000basyony@yahoo.com (M.M.B.); aymanan19@hotmail.com (A.A.H.)

**Keywords:** turmeric extract, ivermectin, sarcoptic mange, performance, antioxidant status, rabbit

## Abstract

**Simple Summary:**

Sarcoptic mange is a common rabbit disease that can be spread directly from sick to healthy rabbits. Infection in rabbits causes intense pruritus, head shaking, and scabby lesions on the inner side of the pinnae and on the external ear canal, as well as diminished growth performance and an increased rate of oxidative stress. Ivermectin has been shown to be an effective treatment for sarcoptic mange in rabbits; however, it has side effects on their performance. Botanicals such as turmeric extract have a remarkable antioxidant potential contributing to the deterrence of *Sarcoptes*-induced oxidative discrepancy in rabbits. The goal of this study was to investigate if turmeric extract can alleviate side effects in Ivermectin-treated rabbits while simultaneously improving their performance and antioxidant status. The results indicated that turmeric extract was utilized with varied efficacy against mites, and also helped rabbits recover faster and improved Ivermectin’s miticidal efficacy by improving performance and compromised immunity. Turmeric extract has strong antioxidant properties, suggesting that it could be used as an adjunctive remedy to reduce the side effects of Ivermectin while treating clinical rabbit sarcoptic mange. Furthermore, no adverse effects were observed in turmeric extract adjunctively supplemented rabbits, and the provided dose regimen of these supplements was found to be safe.

**Abstract:**

In this experiment, the protective effect of turmeric extract (TE) on side effects of Ivermectin-treated rabbits, while improving their performance, blood characteristics, and antioxidant status, was investigated. Sixty-three clinically *Sarcoptes*-infested rabbits aged 60 days were randomly allocated into three groups, with 21 rabbits in each group, to receive either no TE or TE supplementation (1 or 2 mg/kg diet) for 30 days after being subcutaneously injected with Ivermectin (IVM) 1% *w*/*v* at a dose of 0.2 mg/kg body weight twice a week. Another 21 healthy rabbits were used as the control. Treatment with IVM + 1 and 2 mg TE improved body weight (BW), body weight gain (BWG), feed intake (FI), and feed conversion ratio (FCR) in infested rabbits (*p* < 0.05). The IVM alone treatment had the highest mortality rate compared with the other treatments. Rabbits treated with IVM + 1 and 2 mg TE demonstrated progressive recovery manifested by improved nutrient digestibility and nitrogen balance. On day 7, the serum total protein, albumin, and albumin/globulin (A/G) ratio were significantly lower in the treated groups compared with the control group; also, the lowest values were observed in rabbits treated only with Ivermectin, followed by IVM + 1 and 2 mg TE. Treated rabbits had higher thiobarbituric acid reactive substance (TBAR) levels, but lower total antioxidant capacity (TAC), superoxidase dismutase (SOD), and glutathion peroxidase (GSH-Px) levels compared with the control group. On day 30 post-treatment, the rabbits in the IVM + 1 and 2 mg TE treatment groups showed progressive recovery manifested by improved biochemichal parameters, as well as a remarkable improvements in the oxidant/antioxidant balance towards normalcy (*p* < 0.05), and became comparable to that of the control compared with IVM alone treatment. In conclusion, turmeric extract improved rabbits’ performance toward normalcy, and has remarkable antioxidant properties and can be used in conjunction with a miticide to treat *sarcoptic* mange in rabbits.

## 1. Introduction

Sarcoptic mange, caused by the mite *Sarcoptes scabiei*, is a highly contagious, non-seasonal, pruritic skin disorder in rabbits [1], and has the ability to burrow into the skin and feed on both of the epithelial tissues and serum of the host, resulting in severe skin lesions [2]. In addition to causing huge economic losses due to decreased production and increased mortality among animals [3,4], it is an emerging/rejuvenated infectious disease that threatens animal health worldwide [5]. In rabbits, chronic cases of sarcoptic mange can cause anorexia, lethargy, emaciation, and even cause death [6]. Because of their ability to negatively impact the growth rate and feed conversion ratio, sarcoptic mange infestation is one of the major constrains in commercial rabbit rearing [7]. El-Ashram et al. [8] reported that pre-weaning mortality attributable to mite infestation in does was 22.20%. Furthermore, sarcoptic mange contributed to high mortality because does became clinically aggressive as a result of severe irritation and restlessness, which was exacerbated by decreased feed intake, leading to inappetence, weight loss, and poor growth in of kits.

Ivermectin (IVM) has been shown to be an effective treatment for sarcoptic mange in rabbits [9]. Unfortunately, it has a negative effect on rabbit performance, resulting in mild degenerative changes in male rabbits, including complete necrosis of spermatogenic cells with a complete absence of sperms, as well as extreme degeneration and hemorrhage in the uterus, atretic follicles, and degenerated ova in the ovaries of female rabbits [10]. Previous studies have reported that IVM subcutaneously received once every 2 weeks for 2–3 times at a dose of 0.2–0.4 mg/kg of body weight, is usually a simple, safe, and effective treatment against scarcoptic mange [11]. The use of IVM at 400 μg/kg body weight, subcutaneously at weekly intervals for 3 weeks, was also found to be effective at treating scarcoptic mange [12]. However, resistance to currently available IVM treatments has recently been reported in some developing countries [13,14], and as a result of the rise of medication resistance and its harmful consequences in animals, which influence the animal's immune state, nutritional status, and oxidative stress, all of which are predisposing factors for the development of sarcoptic scabies [15,16], researchers are becoming more interested in medical herbs and plant extracts/metabolites [13,17,18]. Thus, the widespread prevalence of *sarcoptic scabies*, combined with declining therapeutic efficacy, necessitates the development of innovative control strategies in the form of adjuvant alternative therapies. 

In this context, antioxidants from natural sources, especially medicinal plant extracts like turmeric extract, have gained much attention. Turmeric (*Curcuma longa*) is a perennial herb that belongs to the ginger family, Zingaberaceae, and is used extensively in Ayurvedic medicine in India. Turmeric contains three different compounds, curcumin, demethoxycurcumin, and methoxycurcumin, all of which are known as curcuminoids [19,20]. Curcumin is responsible for the majority of turmeric’s medicinal properties, including anti-protozoa, antibacterial, antifungal, antiviral, anticancer, anti-inflammatory, antidiabetic, antiproliferative, antioxidant, hypo-cholesterolemic, neuroprotective functions, and hepatoprotective agents [21,22,23]. Turmeric has a wide range of health benefits at a low cost and no has negative effects on livestock production [24]. Due to its vitamin and mineral content, turmeric has also been used as dietary supplementation that improves feed intake, nutrient digestibility, and growth performance in rabbits [25]. Although the efficacy of IVM in treating scabies worldwide has been well established, little is known about its side effects in rabbits [26]. This is because of the paucity of research on the use of natural antioxidants in conjunction with IVM and how the oxidative stress system affects cell susceptibility to this drug. Therefore, the aim of this study was to investigate the effect of the drug when supplemented with known antioxidants from natural sources like turmeric extract (TE) on reducing the drug’s negative effects when used for the treatment of sarcoptic mites in farmed rabbits.

## 2. Materials and Methods

This research was conducted at the Noubaria experimental station’s Rabbit Research Unit, Animal Production Research Institute, the Agricultural Research Centre. The research protocol was permitted by the Animal Care and Use Committees of the Scientific Research and Technological Applications (Protocol No. 27-1W-0521), Alexandria, Egypt.

### 2.1. Preparation of Aqueous Extract of Turmeric

About 500 g of macerated turmeric (*Curcuma longa*) rhizome mash was weighed and mixed in 1 L of distilled water and was allowed to boil for 20 min. The mixture was allowed to stand for 24 h, then the supernatant was removed and introduced in a Buchi rotavapor at 60 °C under a vacuum for sample drying, where 350 mg of aqueous extract was obtained. The obtained concentration of curcumin was 4.5 mg/100 mg of aqueous extract of *Curcuma longa*.

### 2.2. Experimental Design

Sixty-three *Sarcoptes*-infested rabbits were allocated into three groups, each with 21 rabbits, while another group of 21 healthy rabbits served as a control. The rabbits were all 60 days old and weighed about 869.5 ± 94.27 g. The healthy rabbits were free of ectoparasites and helminthes upon microscopic examinations of the skin scraping materials and stool samples. *Sarcoptes*-infested rabbits were treated either with no TE or two TE supplementation doses (1 and 2 mg/kg diet) for 30 days after being subcutaneously injected with a commercial formulation of Ivermectin 1% *w*/*v* at a dose of 0.2 mg/kg body weight twice a week. All rabbits were kept under observation in well-ventilated buildings, and were individually housed in galvanised metal wire cages and maintained under equal managerial, hygienic, and environmental conditions in a room with an ambient temperature of 23 ± 2 °C, humidity 55–65%, and a photoperiod of 16 h light/8 h dark. All cages were equipped with feeding hoppers and drinking nipples, and feed and fresh water were available ad libitum during the experiment. Rabbits were fed a commercial pelleted diet to meet their requirements, according to NRC [27], as shown in Table 1.

### 2.3. Isolation of Mange-Causing Mites

In this experiment, male rabbits infected with *Sarcoptes* (n = 63) were not given any ectoparasiticides or steroid anti-inflammatory medications for 15 days before being examined clinically and dermatologically. Rabbit’s feces were examined for any infection with helminth parasite eggs following the sedimentation technique [29], which relies on the low-density solution into which parasite forms fall. Only scab rabbits with no other ectoparasites were selected in this examination. The dry scraping technique was used to isolate the samples, as described by Fthenakis et al. [5]. Scrapings from infested rabbits were collected from the edges of lesions, particularly within pruritic locations and areas with thick and crusty flakes using a sharp tool at the right angle of the skin and scraping off the outer surface of the skin. The scrapings were deep enough due to mite burrows within the skin layers, as shown by the small amount of blood ooze from the scraping site. Ear mange was collected from scrapings of the yellow-gray skin scales, which was tightly adherent and appeared in the infected ear canal, ear lobes, and infested ear cerumen. The scrapings were then placed in sealed container and sent to the Department of Applied Entomology and Zoology, Faculty of Agriculture, Alexandria University, for microscopic identification and preparation.

### 2.4. Identification of Isolated Mites

The mites were examined under a stereoscopic microscope (Stereomaster II^®^ SPT-ITH, Fisher Scientific, Pittsburgh, PA, USA) and were manually transferred to glass slides containing 2−3 drops of Hoyer’s solution (Mounting fluid). The samples were then covered with glass slide covers and kept in an incubator for 48 h prior to examination. The morphological characteristics of the samples were then examined under a magnification microscope (Leitz^®^ HM-LUX, Stuttgart, Germany) at 40× magnification. Mite identification was performed using the previously described dichotomous key [30,31,32].

### 2.5. Growth Performance

Body weight was recorded at the beginning of the experiment at 60 days of age to determine the initial body weight (IBW), and at the end of the experiment at 90 days of age to determine the final body weight (FBW). Feed intake (FI) was recorded daily during the experiment. The body weight gain (BWG) and feed conversion ratio (FCR) were calculated, and the mortality rate was recorded daily and the percentage was recorded for each group at the end of the experiment.

### 2.6. Digestibility Coefficients and Nitrogen Balance

A digestion and nitrogen balance trial was conducted on 7 rabbits chosen at random from each treatment during the final week of the experiment, according to Perez et al. [33]. Rabbits were housed individually in metabolic cages fitted with a system for collecting feces and urine quantitatively. During the 7-day adaptation period that preceded the main experiment, rabbits were allowed to adapt to the new environment and diet. Pelleted feed in the amount of 120 g was provided once a day in the morning. The rabbits had free access to drinking water. Non-ingested feed residues and feces were collected every day and weighed to a precision of 1 g. The feces and feed samples were oven dried at 60 °C for 48 h, and were ground and analyzed for the dry matter (DM), crude protein (CP), Ether extract (EE), and ash. The CP content (N 6.25) was determined according to Kjeldahl’s method (Method No. 978.04) [33]. The ether extract was determined according to the Soxhlet extract method using petroleum ether as an extract agent (40–60 °C) (Method No. 930.09) [34]. The ash content was assayed by incinerating the samples in a muffle furnace at 550 °C (Method No. 930.05) [34]. The contents of NDF and ADF were analyzed using a Tecator Fibretic System according to the method described by [35]. The nitrogen-free extract (NFE) value was calculated using the difference method. In addition, 10% of the urine collected daily from each animal was preserved for nitrogen determination. The urine of each animal was collected in a glass flask containing 10 mL of 1:1 HCl:H_2_O solution to avoid any bacterial contamination. Nitrogen intake (NI), nitrogen excreted in urine (NU), and nitrogen excreted in the feces (NF) were estimated by the amounts of feed ingested and the excreted nitrogen of the urine and feces. Retained nitrogen (RN) was calculated according to the following equation:

RN = NI − (NF + NU)

### 2.7. Blood Sampling and Biochemical Analysis

On day 7 and 30 of the experiment, five ml of blood samples were obtained from the ear vein of 7 rabbits in each treatment, in clean centrifuge tubes and immediately centrifuged at 3000× *g* for 20 min at 20 °C. Serum was separated and kept at −20 °C for biochemical analyses to determine total protein, albumin, cholesterol, triglyceride, high density lipoprotein (HDL), low density lipoprotein (LDL), aspartate aminotransferase (AST) and alanine aminotransferase (ALT) activity, while globulin was calculated as (total protein−albumin). Blood biochemicals were determined calorimetrically using standard kits supplied by (Bio-diagnostic, Cairo, Egypt) based on the procedure outlined by the manufacturer.

### 2.8. Determination of Antioxidant Indices

The antioxidant enzymes, such as glutathione peroxidase (GPx), superoxide dismutase (SOD), total antioxidant capacity (TAC), and thiobarbituric acid-reactive substances (TBARS) as an index of lipid peroxidation in blood were also determined. The antioxidant indices of blood samples were determined according to the manufacturers’ instructions using commercial assay kits (Bio-diagnostic, Cairo, Egypt).

### 2.9. Statistical Analysis

Data were statistically analyzed using the General Linear Model procedure of the Statistical Analysis System [36]. Data obtained were tested by analysis of variance with one-way design to test the treatment at each sampling according to the following model:

Y_ij_ = μ + D_i_ + ε_ij_

where y_ij_ is the measured value, μ is the overall mean effect, D_i_ is the ith treatment effect, and ε_ij_ is the random error associated with the jth rabbits assigned to the ith treatment. While, data of serum biochemical parameters and serum antioxidants status were analyzed as a randomized complete block design using PROC MIXED of SAS. The obtained data of rabbits were subjected to factorial analysis of variance (2 × 2) according to the following model:

Y_ijk_ = µ + T_i_ + β_j_ + ε_ijk_

where µ is the overall mean, T*_i_* is the effect of the ith level of factor treatment, β_j_ is the effect of the jth level of factor time, and ε_ijk_ is the random error associated with the kth replicate in cell (i, j). Differences among means were considered to be significant at the *p* < 0.05 level, as determined using Duncan’s multiple range test.

## 3. Results

### 3.1. Mite Identity

**Figure 1:**
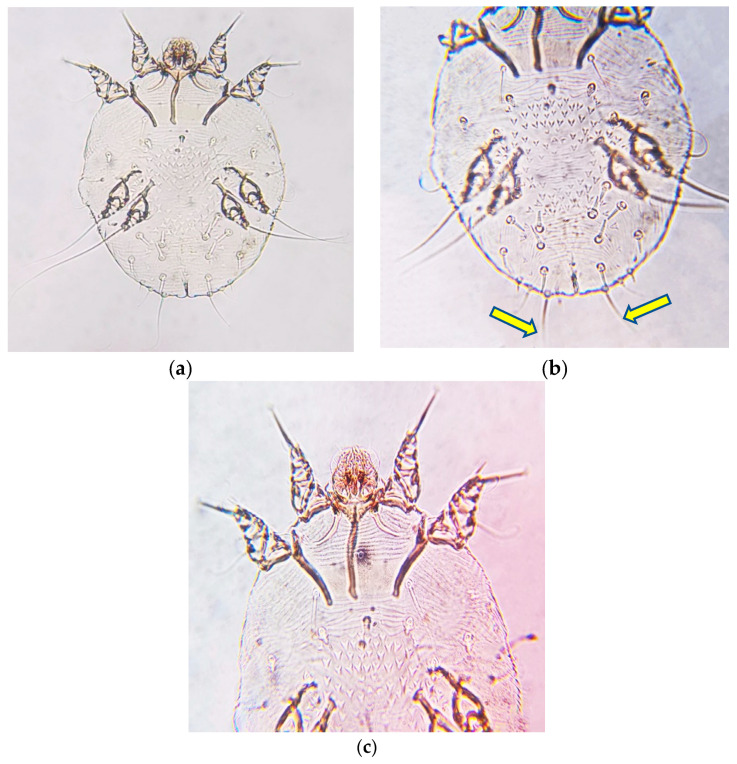
Mite identity: (**a**) Overview of the body shape of the itch mite, *Sarcoptes scabiei* var. *cuniculi*; (**b**) Idiosoma with the cuticular spines in the middle; (**c**) Short capitulum of the itch mite, *Sarcoptes scabiei* var. *cuniculi*.

All morphological descriptions referred to individuals of the itch mite, *Sarcoptes scabiei* var. *cuniculi* (Linnaeus, 1758) (Figure 1). Observably, the idiosoma has an oval tortoise-like shape with cuticular spines and coarse basically in the middle of the idiosoma (Figure 1a), legs III and IV are normally developed and do not extend beyond the lateral-posterior margins of the idiosoma, while, legs I and II extend anteriorly out of the idiosoma, all legs bear and terminate with long setae (Figure 1b), as well as two spur-like claws on leg I, II and III and one on leg IV of the male mites. Gnathosoma has short, stout chelicerae and pedipalps (Figure 1c).

### 3.2. Growth Performance

Both doses of TE inclusion used in the study were effective against the negative effect of IVM alone on rabbit performance during sarcoptic mange treatment, and showed a significant (*p* < 0.05) improvement in growth performance parameters (Table 2). When compared with IVM alone, IVM + 2 mg of TE improved BW, BWG, FI, and FCR in infested rabbits (*p* < 0.05), followed by IVM + 1 mg of TE. The improvement in growth performance parameters in rabbits treated with IVM + 1 mg TE was comparable to that of the control group, with the exception that the BWG in the IVM + 1 mg TE group was higher than in the control group, while the rabbits treated with IVM + 2 mg TE had better growth performance than the other treatments. The group treated with IVM alone had the highest mortality rate followed by the IVM + 1 mg of TE group, while the lowest mortality rate occurred in the IVM + 2 mg of TE group that was similar to that of the control.

### 3.3. Digestbility Coefficients and Nitrogen Balance

By the day 30 post-treatment, the rabbits treated with IVM + 1 and 2 mg of TE demonstrated progressive recovery manifested by improved nutrient digestibility of DM, CP, NDF, and ADF, which was verified by a comparison with the control group (Table 3). DM, CP, NDF, and ADF were all significantly reduced (*p* < 0.05) when injectable IVM was used alone. There were no significant differences in nutrient digestibility between the control group and IVM + 2 mg TE group. Rabbits treated with IVM + 1 and 2 mg of TE demonstrated progressive recovery manifested by improved NI, DN, RN, DN/NI, RN/NI, and RN/DN, which was verified by comparison with the control group on day 30 (Table 4).

### 3.4. Blood Biochemical Constituents

On day 7, in the infested rabbits treated with IVM alone, the total protein, albumin, globulin, and A/G ratio were significantly decreased (*p* < 0.05) (Table 5). The total protein, albumin, and A/G ratio increased in rabbits treated with IVM + 1 and 2 mg of TE compared with the rabbits treated with IVM alone, whereas the globulin levels were similar in the rabbits treated with IVM + 1 and 2 mg of TE and the control group. There were no significant differences in cholesterol, triglyceride, HDL, and LDL among all of the treatments. The concentrations of ALT and AST in the treated rabbits were significantly higher than those in the control on day 7. By day 30 post-treatment, both rabbits in the treatments that received 1 and 2 mg of TE in their diet showed progressive recovery manifested by improved blood biochemistry parameters obtained on day 7, and became comparable to that of the control. However, the rabbits treated with IVM alone could not achieve comparable values to those of the control by day 30 post-treatment.

### 3.5. Blood Serum Antioxidant Constituents

On day 7, infested rabbits treated with either IVM alone or IVM + 1 and 2 mg of TE had significantly higher (*p* < 0.05) TBARS levels, but lower TAC, SOD, and GSH-Px levels when compared with the control (Table 6). The TAC levels in the control rabbits and those treated with IVM + 2 mg TE were similar. By day 30 post-treatment, infested rabbits treated with IVM + 1 and 2 mg of TE displayed progressive recovery, with the oxidant/antioxidant balance remarkably improved towards normalcy (*p* < 0.05). The increased TBARS of these groups was significantly (*p* < 0.05) lowered in comparison with the value obtained on day 7, and this level became comparable to those in the control rabbits by day 30 post-treatment. The levels of TAC, SOD, and GSH-Px were significantly (*p* < 0.05) higher in infested rabbits treated with IVM + 1 and 2 mg of TE when compared with the values obtained on day 7, and were found to be comparable to that of the control rabbits. However, the rabbits treated with IVM alone could not achieve comparable values to those of the control rabbits by day 30 post-treatment.

## 4. Discussion

The results of the present study revealed that *Sarcoptes*-infested rabbits in the IVM alone group had lower body weights, which could be attributed to inappetence, oxidative stress, or inflammation produced by a mite infestation, as well as the influence of IVM treatment. Rabbits may lose their appetite due to dizziness and nausea caused by an ear infection, and because chewing might be painful. Inflammation and infectious diseases markedly alter metabolism and feed utilization. The extent and duration of these metabolic disturbances vary with the type and severity of the disease and, as a consequence, alter growth and delay recovery [37]. Both decreased protein synthesis and sustained increased protein breakdown caused the net catabolism of muscle proteins that produced an increased flow of amino acids from the muscle to the visceral organs [38,39,40]. These amino acids are necessary for energy supply as well as for the processes that are activated as part of the body’s defense mechanisms. Mite infestation and the inflammatory states are often associated with a reduced feed intake, which raises the need for skeletal muscle protein mobilization. A study investigating the causes of mortality in young rabbits [8] reported that pre-weaning mortality attributable to mite infestation in does was 22.20%. Furthermore, sarcoptic mange contributed to high mortality because does became clinically aggressive as a result of severe irritation and restlessness, which was exacerbated by a decreased feed intake leading to inappetence, weight loss, and poor growth in kits. 

Ivermectin has been commonly used to control mite infestation not only in rabbits, but in many other animals. Co-treatment of IVM + 1 and 2 mg TE resulted in a progressive recovery demonstrated by reducing the negative effect of single injectable IVM therapy on the performance and physiological functioning of rabbits. In this context, rabbits fed a diet containing 1.0 g/kg turmeric gained the highest body weight and had the best FCR [25,41]. There was a significant improvement in weight gain when TE was included in the diet at 1 and 2 mg, which may be attributed to its active components with an antioxidant activity, which would stimulate protein synthesis by the rabbit. This finding was similar to that of [42], who found that inclusion of turmeric at 7 g/kg diet affected broiler BWG and resulted in better FCR compared with the other groups. Kumar et al. [43] reported that vitamin supplementation is widely used in conjunction with IVM to improve parasitic and clinical recovery in rabbits infested with the *Sarcoptes scabiei* mite compared with those rabbits treated with only IVM. Thus, administered TE can be used as an adjunctive therapy in rabbits to counteract the negative effects of IVM treatment. Acute phase reactions are triggered by parasitism and/or the infection of animals by any pathogen, and they comprise both physiological and metabolic alterations [44]. 

In this study, ear inflammation and abnormal skin surface were reported in infested rabbits, and these symptoms led to a nitrogen imbalance. Tissue protein breakdown might result in a negative nitrogen balance if adequate and optimal nutrition is not provided [45]. Notably, our findings indicated that a negative nitrogen balance occurred more in infested rabbits treated with IVM alone owing to the low feed intake and low protein oxidation rates, which resulted in a hypo-metabolic pattern, whereas for infested rabbits treated with IVM + 1 or 2 mg TE it improved their nitrogen balance compared with the control rabbits. This improvement is attributed to the polyphenol curcumin, which is extracted from dried plant rhizomes and inhibits mitogen-activated protein kinases, as reported by Jeon et al. [46]. Plant extracts contain a variety of molecules with bioactivities that are specific to the physiology and metabolism of animals [47]. Various medicinal plant extracts have been proposed to have appetite-stimulating, digestive-stimulating, and antimicrobial properties [48]. According to our findings, TE may increase bile production and excretion in the small intestine, resulting in increased fat, protein, and carbohydrate digestion. The combination of IVM + TE improved the performance of infected rabbits. This could be due to active components that increase the digestion and absorption of nutrients, or it could be due to lower peroxidation. Curcuminoids and curcumin of turmeric were found to increase feed utilization, resulting in an improved growth performance [42]. According to Zeweil et al. [25], feeding rabbits a diet supplemented with 1g of turmeric/kg increased the digestibility of DM and CP compared with the control. It was shown that TE at a dose of 0.04 mL/kg of body weight had the best digestibility coefficient for OM and CP compared with the control group [49]. 

Many factors can affect albumin circulation in the blood, including chronic liver disease, which can lower albumin levels in the blood and parasitism by ecto-or endo-parasites, which can cause inflammation and necrosis in the animals’ livers, resulting in a decrease in albumin production [50]. Certain medicinal plant extracts, such as TE, contain several bioactive components like curcumin, curcuminoids, and other polypeptides. These components have antioxidant, anti-inflammatory, and anti-proliferative properties, and some have been used as hepatoprotective agents [22], suggesting that they can boost the development of such proteins, especially in parasitism or IVM therapy. The administration of IVM + 1 or 2 mg TE to infested rabbits increased the total protein, albumin, and A/G ratio compared with those rabbits treated with IVM alone. Several studies have previously shown the importance of combining IVM with other treatments to suppress mites, such as neem extract [51], garlic and cinnamon oil [52], and garlic therapy [53]. These treatments have been shown to increase the protein, albumin, and globulin levels compared with the untreated animals. Zeweil et al. [25] observed that different doses of turmeric (0.5 and 1.0 g/kg) significantly decreased triglycerides and LDL compared with the control group. In addition, [42] found that increasing turmeric levels to 7 g/kg diet significantly decreased the triglycerides and total cholesterol. Curcumin, which enhances bile production and therefore lipid digestion, may be responsible for the lower levels of triglycerides, total cholesterol, and LDL [54]. The levels of liver enzymes such as ALT and AST can be measured as indicators of any hepatocyte damage that may occur as a result of parasitism-induced liver necrosis and inflammation or hepatic lipidosis [50]. Our findings revealed a significant elevation of ALT and AST levels in the rabbits treated with either IVM alone or treated with IVM + 1 and 2 mg TE/kg diet on the day 7 post-treatment, which may be due to damage to rabbit liver tissues caused by mite infestation and toxic excretory products. Similar results have been observed previously with other treatments [51,55]. Ivermectin’s deleterious impact on rabbit livers is confirmed by this increase in ALT and AST levels, and similar relationships have been reported previously in the case of macrocyclic lactones [56]. In general, the toxic effect of IVM on the liver function is fairly long-term and takes at least 3 months for the effects to disappear and for the animals to return to their normal state [56,57]. However, by day 30 post-treatment, rabbits treated with IVM + TE showed a significant (*p* < 0.05) amelioration in the aforementioned serum biochemistry towards normalcy more rapidly compared with the rabbits treated with IVM alone. 

*Sarcoptes*-infested rabbits in the IVM alone group were found to be in a state of severe oxidative stress; this result is consistent with previous scientific reports that have related altered antioxidant systems to states of oxidative stress in animals with parasitic skin infestations, such as sarcoptic mange [18]. Amelioration of the altered oxidant/antioxidant balance towards normalcy in IVM + 1 and 2 mg of TE treated rabbits indicates a potential antioxidant action of the adjunctive treatments. By day 30, the administration IVM + 1 or 2 mg TE to infested rabbits significantly improved the decrease of TAC, SOD, and GSH-Px levels back towards normalcy. The results of this study indicated that the presence of a large quantity of antioxidant compounds (flavonoids and polyphenols) in TE may have resulted in a remarkable amelioration of oxidative stress caused by sarcoptic mange. TE supplementation to infested rabbits during IVM injection therapy may accelerate the altered redox/antioxidant balance towards normalcy and provide antioxidant restoration, and improved lipid peroxidation could play a role in faster clinical recovery in treated rabbits. Singh et al. [58] reported that adjunct therapy with antioxidants resulted in a faster clinical recovery and a transition in oxidative imbalance toward normalcy in rabbits infected with *Psoroptes cuniculi*. By day 30 post-treatment, TE may have a complementary impact with IVM injection as an antioxidant defense system of *Sarcoptes*-infested rabbits, significantly improving the healing effect of IVM and avoiding IVM’s unfavorable effects on rabbit performance and physiological function. TE indirectly enhances the miticidal effect of IVM due to a variety of factors, including immunomodulatory and antioxidant properties, which help the animal’s body fight off *Sarcoptes* mites.

## 5. Conclusions

Turmeric extract has a remarkable antioxidant potential contributing to the deterrence of *Sarcoptes*-induced oxidative discrepancy in rabbits. It can also help rabbits recover faster and improve Ivermectin’s miticidal efficacy by improving performance and compromised immunity. Furthermore, no adverse effects were observed in turmeric extract adjunctively supplemented rabbits, and the provided dose regimen of these supplements was found to be safe. The findings recommend turmeric extract as an adjunctive remedy along with the miticide (Ivermectin) to treat clinical rabbit’s sarcoptic mange. Large-scale research is also required to confirm these assumptions.

## Figures and Tables

**Table 1:** Ingredients and chemical composition of the experimental diet (% DM basis).

Ingredients	(%)
Soybean meal (44% CP)	20.9
Barley	32.0
Wheat bran	9.2
berseem hay	31.0
Molasses	3.0
Limestone	0.7
Di-Ca-phosphate	2.2
DL-Methionine	0.4
NaCl	0.3
Vit.-Min. premix *	0.3
Total	100
Chemical composition (% DM basis):	
Dry matter	91.00
Organic matter	83.38
Crude protein	17.20
Crude fibre	12.90
Ether extract	2.60
Nitrogen free extract	58.30
Ash	7.62
Neutral detergent fibre	37.27
Acid detergent fibre	26.83
DE * (Kcal/Kg DM)	2530

* Mineral and vitamin mixture supplied per kg of diet: Vitamin A, 10,000 IU; Vitamin D3, 1800 UI; Vitamin E, 15 mg; Vitamin K3, 4.5 mg; Vitamin B1, 0.5 mg; Vitamin B2, 4 mg; Vitamin B12, 0.001 mg; Folic acid, 0.1 mg; Pantothenic acid, 7 mg; Nicotinic acid, 20 mg; I, 1 mg; Mn, 60 mg; Cu, 5.5 mg, Zn, 75 mg; Fe, 40 mg; Co, 0.3 mg; Se, 0.08 mg; Robenidine, 52.8 mg, Antioxidant, 0.250 mg. * DE: digestible energy (Kcal/KgDM) = TDN × 44.3, according to [28].

**Table 2:** The effect of combining of Ivermectin injection with turmeric extract supplementation on rabbit growth performance.

Treatments	Growth Performance					
	IBW (g)	FBW (g)	BWG (g/d)	FI (g/d)	FCR	Mortality %
Control	918.8	1934.7 ^b^	33.9 ^b^	96.9 ^a^	2.86 ^b^	4.76 ^c^
IVM	860.8	1527.1 ^c^	22.1 ^c^	84.1 ^b^	3.81 ^a^	19.05 ^a^
IVM + 1 mg TE	841.1	1929.3 ^b^	36.3 ^a^	99.4 ^a^	2.74 ^bc^	9.52 ^b^
IVM + 2 mg TE	857.3	1996.8 ^a^	38. 0 ^a^	96.1 ^a^	2.53 ^c^	4.76 ^c^
SEM	81.25	47.01	1.73	14.06	0.19	2.46
*p*-value	0.859	0.001	0.003	0.027	0.013	0.001

^a–c^ Means within a column with different superscripts are significantly different (*p* < 0.05); IBW, Initial body weight; FBW, final body weight; BWG, body weight gain; FI, feed intake; FCR, feed conversion ratio. IVM, Ivermectin; TE, turmeric extract. Number of observation = 84, with 21 rabbits/group.

**Table 3:** The effect of combining of Ivermectin injection with turmeric extract supplementation on growing rabbit digestibility coefficients.

Treatments	Digestibility Coefficients			
	DM %	CP %	NDF %	ADF %
Control	63.77 ^a^	62.41 ^a^	54.26 ^a^	51.44 ^a^
IVM	57.87 ^c^	58.37 ^c^	37.05 ^c^	35.78 ^c^
IVM + 1 mg TE	62.35 ^b^	61.04 ^b^	52.87 ^b^	49.71 ^b^
IVM + 2 mg TE	63.41 ^a^	62.16 ^a^	54.37 ^a^	51.26 ^a^
SEM	0.33	0.17	0.22	0.16
*p* value	0.001	0.002	0.001	0.001

^a–c^ Means within a column with different superscripts are significantly different (*p* < 0.05). DM, dry matter; CP, crude protein; NDF, neutral detergent fiber; ADF, acid detergent fiber. IVM, vermectin; TE, turmeric extract. Number of observations = 84, with 21 rabbits/group.

**Table 4:** The effect of combining Ivermectin injection with turmeric extract supplementation on the nitrogen balance of growing rabbits.

Treatments	Nitrogen Balance							
	NI g/d	FN g/d	UN g/d	DN g/d	RN g/d	DN/NI %	RN/NI %	RN/DN %
Control	2.66 ^a^	0.69 ^b^	0.55 ^b^	1.97 ^a^	1.42 ^a^	74.06 ^a^	53.38 ^a^	72.08 ^a^
IVM	2.40 ^b^	0.79 ^a^	0.73 ^a^	1.61 ^b^	0.88 ^b^	67.08 ^b^	36.67 ^b^	54.66 ^b^
IVM + 1 mg TE	2.68 ^a^	0.66 ^b^	0.54 ^b^	2.02 ^a^	1.48 ^a^	75.37 ^a^	55.22 ^a^	73.27 ^a^
IVM + 2 mg TE	2.63 ^a^	0.68 ^b^	0.53 ^b^	1.95 ^a^	1.42 ^a^	74.14 ^a^	53.99 ^a^	72.82 ^a^
SEM	0.14	0.04	0.02	0.11	0.14	1.04	1.96	1.22
*p* value	0.004	0.006	0.035	0.022	0.008	0.001	0.012	0.001

^a,b^ Means within a column with different superscripts are significantly different (*p* < 0.05). NI, Nitrogen intake g/d; FN, Fecal N g/d; UN, urinary N g/d; DN, digestible N g/d; RN, retained N g/d; DN = NI − FN, RN = NI − FN − UN, DN/NI (%), the efficiency of N intake converted into digestible N; RN/NI (%), the efficiency of N intake converted into retained N; RN/DN (%), the efficiency of digestible N converted into digestible N; IVM, Ivermectin; TE, turmeric extract. Number of observations = 84, with 21 rabbits/group.

**Table 5:** The effect of combining Ivermectin injection with turmeric extract supplementation on the serum biochemical parameters.

Treatments	Blood Parameters										
	Day	TP mg/dL	ALB mg/dL	GLOB	A/G Ratio	TG mg/dL	CHO mg/dL	HDL mg/dL	LDL mg/dL	ALT U/L	AST U/L
Control	7	6.34 ^a^	3.66 ^a^	2.68 ^a^	1.37 ^a^	48.66	88.92	29.91	15.05	31.05 ^b^	31.51 ^b^
	30	6.31 ^a^	3.51 ^a^	2.60 ^a^	1.35 ^a^	53.10	99.02	33.65	15.94	33.86 ^b^	29.50 ^b^
IVM	7	4.91 ^c^	2.51 ^c^	2.40 ^b^	1.05 ^b^	47.91	89.1	30.44	14.49	42.75 ^a^	37.66 ^a^
	30	5.05 ^b^	2.70 ^c^	2.35 ^b^	1.15 ^b^	47.24	89.17	32.63	14.45	44.64 ^a^	39.14 ^a^
IVM + 1 mg TE	7	5.80 ^b^	3.14 ^b^	2.66 ^a^	1.18 ^b^	51.16	103.68	25.06	15.19	44.01 ^a^	39.06 ^a^
	30	6.33 ^a^	3.69 ^a^	2.64 ^a^	1.40 ^a^	46.45	97.83	30.25	13.96	31.43 ^b^	25.76 ^b^
IVM + 2 mg TE	7	5.90 ^b^	3.19 ^b^	2.71 ^a^	1.18 ^b^	46.70	79.67	29.29	14.38	44.23 ^a^	38.88 ^a^
	30	6.36 ^a^	3.64 ^a^	2.68 ^a^	1.37 ^a^	51.3	90.61	30.14	1369	34.06 ^b^	28.67 ^b^
SEM		0.07	0.21	0.10	0.10	6.17	16.01	4.73	2.56	3.84	4.62
*p-*value		0.001	0.011	0.01	0.041	0.844	0.792	0.572	0.663	0.031	0.005

^a–c^ Means within a column with different superscripts are significantly different (*p* < 0.05). TP, total protein; ALB, total albumin; GLOB, globulin; A/G ratio, albumin to globulin ratio; CHO, cholesterol; TG, triglycerides; HDL, high density lipoprotein; LDL, low density lipoprotein; ALT, alanine aminotransferase; AST, aspartate aminotransferase; IVM, Ivermectin; TE, turmeric extract. Number of observations = 84, with 21 rabbits/group.

**Table 6:** The effect of combining Ivermectin injection with turmeric extract supplementation on the serum antioxidants status.

Treatments	Blood Antioxidants Parameters				
	Day	TBARS (U/mL)	TAC (U/mL)	SOD (U/mL)	GSH-Px (mU/mg)
Control	7	4.36 ^b^	2.29 ^a^	23.66 ^a^	537.1 ^a^
	30	4.37 ^b^	2.30 ^a^	23.78 ^a^	541.3 ^a^
IVM	7	6.01 ^a^	1.77 ^b^	19.11 ^b^	256.8 ^c^
	30	5.71 ^a^	1.87 ^b^	20.15 ^b^	355.6 ^c^
IVM + 1 mg TE	7	6.11 ^a^	1.79 ^b^	18.70 ^b^	344.8 ^b^
	30	4.25 ^b^	2.45 ^a^	24.36 ^a^	539.9 ^a^
IVM + 2 mg TE	7	6.36 ^a^	2.05 ^a^	18.65 ^b^	356.2 ^b^
	30	3.18 ^b^	2.61 ^a^	23.71 ^a^	602.1 ^a^
SEM		0.07	0.10	0.19	0.85
*p-*value		0.01	0.05	0.03	0.004

^a–c^ Means within a column with different superscripts are significantly different (*p* < 0.05). TBAR, thiobarbituric acid reactive substance; TAC, total antioxidant capacity; SOD, superoxidase dismutase; GSH-Px, glutathion peroxidase; IVM, Ivermectin; TE, turmeric extract. Number of observations = 84, with 21 rabbits/group.

## Data Availability

The data presented in this study are available on request from the corresponding author.
